# Comparative Study of Early Impacts of Post-COVID-19 Pneumonia on Clinical Manifestations, Pulmonary Function, and Chest Radiographs

**DOI:** 10.3390/medicina58020216

**Published:** 2022-02-01

**Authors:** Nutchanok Niyatiwatchanchai, Athavudh Deesomchok, Warawut Chaiwong, Pilaiporn Duangjit, Chaicharn Pothirat, Chalerm Liwsrisakun, Chaiwat Bumroongkit, Theerakorn Theerakittikul, Atikun Limsukon, Pattraporn Tajarernmuang, Konlawij Trongtrakul, Juntima Euathrongchit, Yutthaphan Wannasopha, Tanop Srisuwan

**Affiliations:** 1 Division of Pulmonary, Critical Care and Allergy, Department of Internal Medicine, Faculty of Medicine, Chiang Mai University, 110 Inthawaroros Road Sriphum, Chiang Mai 50200, Thailand; nutchanok.n@cmu.ac.th (N.N.); warawut.chai@cmu.ac.th (W.C.); pilaiporn.th@cmu.ac.th (P.D.); chaicharn.p@cmu.ac.th (C.P.); chalerm.liw@cmu.ac.th (C.L.); chaiwat.b@cmu.ac.th (C.B.); theerakorn.t@cmu.ac.th (T.T.); atikun.limsukon@cmu.ac.th (A.L.); pattraporn.t@cmu.ac.th (P.T.); konlawij.tr@cmu.ac.th (K.T.); 2 Department of Radiology, Faculty of Medicine, Chiang Mai University, Chiang Mai 50200, Thailand; juntima.eua@cmu.ac.th (J.E.); yutthaphan.w@cmu.ac.th (Y.W.); tanop.s@cmu.ac.th (T.S.)

**Keywords:** COVID-19, lung function, exercise capacity, chest radiography, pneumonia, mood disorders

## Abstract

*Background and Objectives*: Scant data regarding early post-COVID-19 effects are available, especially in younger people. Therefore, the objective of this study was to explore the early clinical impacts of post-COVID-19 pneumonia, comparing severe and non-severe patients. *Materials and Methods*: A cross-sectional study was conducted in adult patients admitted with COVID-19 pneumonia from April to May 2021. Demographic data, symptoms and signs, quality of life, Hospital Anxiety and Depression Scale (HADS), chest radiograph (CXR), pulmonary function tests (spirometry, impulse oscillometry), fractional exhaled nitric oxide (FeNO), and exercise capacity were assessed one month after hospital discharge. Twenty-five healthy control subjects that were age- and gender-matched were recruited for comparisons. *Results*: One hundred and five patients, with a mean age of 35.6 ± 15.8 years and 54 (51.4%) males, participated and were categorized into the non-severe pneumonia (N = 68) and severe pneumonia groups (N = 37). At a one-month follow-up visit (the time from the onset of the disease symptoms = 45.4 ± 5.9 days), the severe group had more cough, fatigue, and skin rash with higher dyspnea scale, more residual CXR lesions, and lower quality of life scores. Forced vital capacity (FVC) was lower in the severe group (88.3% of predicted value) and non-severe group (94.6% of predicted value) than in the healthy controls (*p* = 0.001). The six-minute walk distance was significantly lower in the non-severe group, at 79.2 m, and in the severe group, at 103.8 m, than in the healthy control subjects (*p* < 0.001). *Conclusions*: Adult patients with COVID-19, especially those with clinically severe pneumonia, still had residual symptoms and chest radiographic abnormalities, together with poorer quality of life and lower exercise capacity, one month after hospital discharge.

## 1. Introduction

On 31 December 2019, cases of pneumonia caused by novel coronavirus SARS-CoV-2 (COVID-19 pneumonia) were reported from Wuhan city Hubei Province of China [1]. This virus outbreak spread to other countries, affecting nearly 200 million people, and was responsible for over 4 million deaths worldwide as of July 2021 [2]. In Thailand, 597,287 people were infected, and 4857 people died by the end of July 2021 [3]. In April 2021, phase 3 of the COVID-19 outbreak began in Chiang Mai, Thailand, especially among young adults, and infected more than one hundred people per day. At the end of May 2021, there were 4068 cases of COVID-19 in Chiang Mai [3].

The respiratory tract is the most common site of COVID-19 infection, with common symptoms including fever, cough, sputum production, fatigue, shortness of breath, myalgia, sore throat, rhinorrhea, and headache [4,5]. The World Health Organization (WHO) categorizes the clinical severity of COVID-19 as asymptomatic, mild, moderate (non-severe pneumonia), severe (severe pneumonia), and critical disease [6]. In cases of COVID-19 pneumonia, most of the chest radiographs (CXR) have shown ground-glass opacification (GGO), consolidation, or combination with bilateral lower lobe involvement [7].

Cohort studies in patients who have recovered from COVID-19 pneumonia have shown reduced health-related quality of life (HR-QoL) in all domains at two months after hospital discharge [8], with reduced physical activity and exercise performance and mild depression and anxiety at six weeks after hospital discharge [9]. These abnormalities might be related to abnormal lung function and might affect their performance [10]. Studies of CXR findings showed that many patients had imaging abnormalities after hospital discharge, such as residual GGO and pulmonary fibrosis [8,11]. The post-COVID-19 sequelae can affect various organs and can be explained by ongoing chronic inflammation and tissue damage [12]. However, patients with COVID-19 pneumonia in our setting were younger than in previous studies [8,9,11]. Therefore, we sought to explore the early impacts of post-COVID-19 pneumonia, including clinical manifestations, quality of life, mood disorders, pulmonary function tests, exercise capacity, FeNO, and CXR findings, among severe and non-severe COVID-19 pneumonia patients.

## 2. Materials and Methods

### 2.1. Study Design

This cross-sectional study was approved by the Research Ethics Committee, Faculty of Medicine, Chiang Mai University (Study code: MED-2564-08109, date of approval: 3 May 2021) and filed under the Clinical Trials Registry (Study ID: TCTR20210827005, date of approval: 27 August 2021) in compliance with the Declaration of Helsinki. Written informed consent was obtained from all participants. Data collection was conducted in subjects aged over 18 years old, with COVID-19 pneumonia, who were admitted during April–May 2021 and followed up one month after discharge from Maharaj Nakorn Chiang Mai Hospital, Chiang Mai, Thailand. Subjects who were unable to understand the Thai language were excluded. COVID-19 pneumonia was diagnosed by clinical symptoms and signs, pulmonary infiltration on CXR, and confirmed by positive reverse transcription polymerase chain reaction (RT-PCR). These patients were usually treated in our hospital for two weeks or until clinical improvement according to the current clinical practice guidelines of the Ministry of Public Health, Thailand [13]. Baseline demographics, patients’ medical records, and CXR during hospitalization were reviewed by radiologists. Symptoms, HR-QoL questionnaires, including the 36-item Short-Form Health Survey (SF-36) and Euro Quality of Life—5 Dimensions—5 Levels (EQ-5D-5L), Hospital Anxiety and Depression Scale (HADS), pulmonary function tests, exercise capacity, FeNO, and CXR were assessed at the follow-up visit.

### 2.2. Data Collection

Clinical manifestations were assessed on various aspects including clinical symptoms and physiological signs, quality of life (SF-36 and EQ-5D-5L), and major mood disorders (anxiety and depression). The SF-36 questionnaire measures general health status and comprises 36 questions that cover eight domains: physical functioning, role limitations due to physical health problems, role limitations due to emotional problems, vitality (energy and fatigue), emotional well-being, social functioning, bodily pain, and general health perceptions. Each domain has a score ranging from 0 to 100, where a higher score indicates better health [14].

The EQ-5D-5L questionnaire measures health status and consists of the EQ-5D descriptive system and EQ visual analog scale (VAS). The EQ-5D descriptive system comprises five dimensions: mobility, self-care, usual activities, pain/discomfort, and anxiety/depression, where each dimension has five levels, from no problems to slight, moderate, severe, and extreme problems. The score ranges from 0 to 1, in which a score close to 1 shows better quality of life. EQ-VAS records the subject’s self-rated health on a vertical scale from 0 to 100, with the endpoints labeled “The best health you can imagine” and “The worst health you can imagine” [15].

The Hospital Anxiety and Depression Scale (HADS) questionnaire measures psychological distress resulting from a disease and contains 14 items with two subscales: anxiety and depression. Each item has a score of 0–3, giving a maximum score of 21 for anxiety and depression. A score of 11 or higher on either subscale is considered to indicate the probable presence of anxiety or depression, respectively [16].

Chest radiograph was evaluated and scored by radiologists. Several studies have demonstrated the association of scores and clinical manifestations, rate of ICU admission, and death [17,18]. The scoring method in this study was modified and adjusted by dividing the bilateral lung into three zones: the upper lung zone (area above aortic arch), the middle lung zone (area between aortic arch and the inferior margin of the left pulmonary hilum), and the lower lung zone (area below left hilum). The total score of six areas was a summation of each lung zone that was calculated by the multiplication point of the involved area with a density of opacity (0 point—lung involvement of 0%; 1, 2, 3, and 4 points—lung involvement in the range of 1–25%, 26–50%, 51–75%, and 76–100%, respectively) and density of opacity (0 points—no opacity, 1 point—ground-glass opacity, and 2 points—consolidation). The total scores were summarized and ranged from 0 to 48 [19,20].

All subjects were assessed for pre-bronchodilator pulmonary function tests including impulse oscillometry (IOS) and spirometry. IOS was performed before spirometry. IOS and spirometry were performed using combined IOS and spirometry equipment (MostGraph-02; Chest M.I., Co Ltd., Tokyo, Japan).

For IOS measurement, the subjects were asked to perform tidal breathing for 30–40 s via a mouthpiece that was connected to a loudspeaker that generated pressure oscillations composed of multiple frequencies. A minimum of three tests was performed, following the European Respiratory Society (ERS) standard [21]. The average values from three IOS measurements were recorded. We collected the following IOS parameters: airway resistance including resistance at 5 Hz (R5), resistance at 20 Hz (R20), heterogeneity of resistance (R5–R20), and airway reactance including reactance at 5 Hz (X5); resonant frequency (Fres); and area under reactance curve between 5 Hz and Fres (AX) [16]. The cut-off point of R5–R20 0.1 kPa/L/s or higher was defined as the presence of small airway disorder [22].

For spirometry assessment, a minimum of three acceptable tests was performed, following the American Thoracic Society/European Respiratory Society (ATS/ERS) guidelines [23]. The spirometry parameters, including forced vital capacity (FVC), forced expiratory volume in the first second (FEV_1_), the ratio of FEV_1_/FVC, and forced expiratory flow at 25–75% of FVC (FEF_25–75%_), were measured. Predicted values were calculated using the Global Lung Function Initiative (GLI) reference equation (Southeast Asian population) [24].

The FeNO was performed before IOS and spirometry using FeNO equipment (NIOX VERO^®^ Circassia Inc., Morrisville, NC, USA). The FeNO was performed to evaluate airway inflammation according to the ATS/ERS guidelines [25]. A FeNO value higher than 25 ppb indicated a high possibility of eosinophilic airway inflammation [26].

The six-minute walk test (6-MWT) is a simple practical test measuring the distance that subjects can quickly walk in six minutes. The 6-MWT was performed following the instructions of the ATS [27]. The exercise desaturation was classified as a decrease of 3% or higher in oxygen saturation via pulse oximetry (SpO_2_) between resting and post 6-MWT [28].

Twenty-five age- and gender-matched healthy control subjects with no history of COVID-19 or other respiratory diseases were recruited to this study. Demographic data, symptoms, and signs, CXR, SF-36, ED-5Q-5L, HADS, IOS, spirometry, FeNO, and six-minute walking distance (6-MWD) were collected for comparison with COVID-19 pneumonia subjects.

After obtaining the consent of the ethics committee to record the data from all subjects, a database using Microsoft Excel was organized. In this database, the personal data of the patients were adequately encoded to guarantee data protection.

### 2.3. Study Size Estimation

Sample size calculation was based on the mean and SD of 6-MWD at one month after discharge between severe COVID-19 and non-severe COVID-19 in the previous study [11]. The means and SD of 6-MWD in severe COVID-19 and non-severe COVID-19 was 517.43 ± 44.55 m and 573.52 ± 38.38 m, respectively. We needed to study at least 20 subjects, 10 non-severe COVID-19 and 10 severe COVID-19, to be able to reject the null hypothesis that the population means of the severe and non-severe groups were equal with probability (power) of 0.8. The type I error probability associated with this test of the null hypothesis was 0.05.

### 2.4. Statistical Analysis

Continuous data were expressed as mean and standard deviation (SD), or median and interquartile range (IQR). Analysis of variance (ANOVA) with Bonferroni adjustment was used for analyzing clinical characteristics across the three groups for continuous data. The Kruskal–Wallis test was used for analyzing clinical characteristics across the three groups for non-parametric data. The Student t-test or Mann–Whitney U test was used for analyzing clinical characteristics between severe and non-severe groups for parametric and non-parametric data, respectively. Categorical data were expressed as frequencies and percentages. Comparison of categorical data between groups was performed using Fisher’s exact test. Gaussian regression adjusted for confounding factors including age, body mass index (BMI), and gender was used for analyzing differences in mean values of IOS parameters and 6-MWD across the three groups. A generalized linear model adjusted for confounding factors including age, BMI, and gender was used for analyzing differences in the proportion of exercise desaturation in 6-MWT between severe and non-severe groups. A *p*-value less than 0.05 was considered statistically significant. All statistical analyses were performed using STATA version 16 (StataCorp, College Station, TX, USA).

## 3. Results

**Figure 1:**
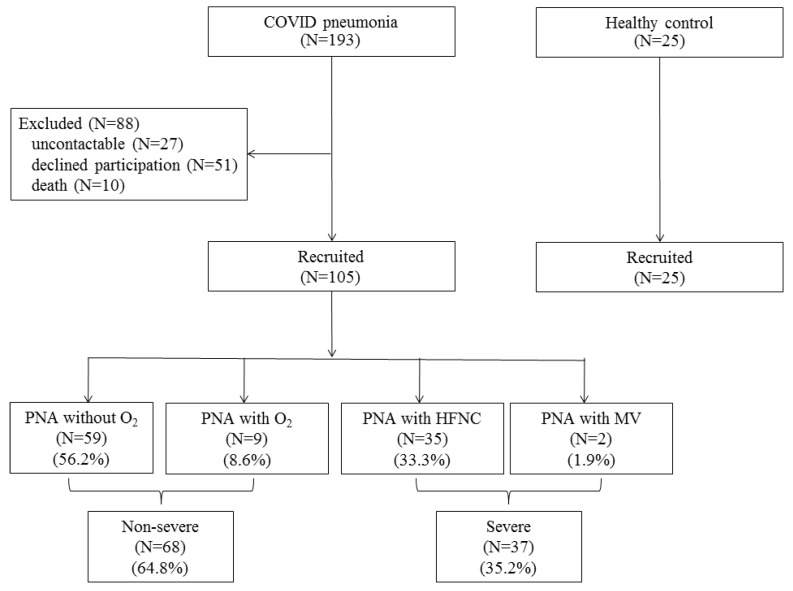
Flow chart of the study population. Abbreviations: PNA, pneumonia; HFNC, high-flow nasal cannula oxygen; MV, mechanical ventilation.

During the phase 3 (April–May 2021) outbreak of COVID-19 in Chiang Mai, 193 patients with COVID-19 pneumonia were admitted to our hospital and ten patients (5.2%) died during hospitalization. One hundred and five discharged patients came for follow-up and participated in this study, with a mean age of 35.6 ± 15.7 years and with 54 males (51.4%). These patients were categorized into two groups: the non-severe pneumonia group, N = 68 (pneumonia with no oxygen therapy, N = 59, or treated with low-flow oxygen cannula, N = 9), and severe pneumonia group, N = 37 (pneumonia treated with high-flow nasal cannula oxygen (HFNC), N = 35, or mechanical ventilator, N = 2). Twenty-five age- and gender-matched healthy control subjects were also enrolled (Figure 1). The demographic data of the study population are shown in Table 1. The non-severe pneumonia patients were younger than the severe pneumonia patients, while the severe pneumonia patients had a higher BMI and had more cardiovascular co-morbidities, where hypertension was the most common (N = 16, 43.2%), compared to the non-severe pneumonia patients. More data are shown in Table 1.

The data collected during hospitalization for COVID-19 pneumonia are shown in Table 2. The severe group had greater clinical severity, with a higher neutrophil–lymphocyte ratio (NLR), platelet–lymphocyte ratio (PLR), and c-reactive protein (CRP) level than the non-severe group. Most of the CXR pattern was GGO (N = 71, 67.6%) with multi-lobar involvement (N = 89, 84.8%) and lower lung zone predominance (N = 102, 97.1%). The CXR score in the severe group was higher, with more consolidation and mixed with GGO than the non-severe group. Remdesivir, empirical antibiotics, systemic corticosteroids, and more aggressive treatments, such as tocilizumab and hemoperfusion, were more frequently prescribed in the severe group. More data are shown in Table 2.

At the one-month follow-up visit, the mean time of the onset of the disease symptoms to follow-up visit was 45.4 ± 5.9 days. Fifty subjects (47.6%) had at least one symptom, with a higher frequency in the severe group (N = 24, 64.9%). In the severe group, cough, fatigue, and skin rash were the most common symptoms, with a higher dyspnea scale and lower oxygen saturation (Table 3).

Health-related quality of life, measured by EQ-5D-5L and SF-36, was also significantly lower in the severe group compared to the non-severe and healthy control groups. In the severe group, quality of life was significantly worse in all domains. Only a few patients (N = 7, 6.7%) had anxiety and/or depression but there was no significant difference between groups. More data are shown in Table 4. In the severe group, CXR revealed more residual lesions with a higher score than the in non-severe group, while NLR and PLR decreased to values closer to the non-severe and healthy control groups (Table 5). Pulmonary embolism was diagnosed in two patients in the severe group during follow-up.

Pulmonary function tests including spirometry and IOS, FeNO, and 6-MWT were assessed in 85 patients (31 patients in the severe group, 29 patients in the non-severe group, and 25 healthy control subjects). The %predicted of FVC was significantly lower in the severe and non-severe groups, and the %predicted of FEV_1_ was also significantly lower in the severe group compared to the healthy controls. Only one patient in the severe group had an obstructive defect defined by FEV_1_/FVC < LLN. The FeNO level was not significantly different across the three groups. The proportion of FeNO > 25 ppb was more frequent in the non-severe group compared to the severe and healthy control groups, but not statistically significant. More data are shown in Table 6.

IOS parameters were also not significantly different across the three groups, except for R20, which was significantly lower in the non-severe group than healthy controls. Ten patients in the severe group (32.3%), eight patients in the non-severe group (27.6%), and nine healthy control subjects (36.0%) had evidence of small airway disorder defined by R5-R20 > 0.1 kPa/L/s. More data are shown in Table 7.

Subjects in the severe and non-severe groups had significantly lower 6-MWD compared to healthy controls. Subjects in the severe group had more oxygen desaturation during 6-MWT than the non-severe group, but this was not statistically significant (adjusted risk ratio = 3.31 (95%CI: 0.38, 28.96)). More data are shown in Table 8.

## 4. Discussion

We studied the impacts of COVID-19 pneumonia on clinical manifestations including HR-QoL and psychological problems, pulmonary function, exercise capacity, FeNO, and chest radiograph at a one-month follow-up visit after hospitalization for COVID-19 pneumonia and found that patients with COVID-19 pneumonia, especially clinically severe patients, still had more symptoms, including dyspnea, worse quality of life, lower exercise capacity, and more residual CXR lesions. Although the spirometric parameters of these post-COVID-19 patients were within normal limits, there was a trend of lower lung volume with increased severity of pneumonia, similar to previous studies [8,9,11].

This study found that cough, fatigue, and skin rash were significantly more frequent in the severe COVID-19 pneumonia group than the non-severe group. Cough and fatigue may be affected by the residual inflammatory process. During the acute phase of COVID-19, our patients with severe pneumonia had more lung inflammation, demonstrated by higher CXR scores, as well as more systemic inflammation, demonstrated by increased inflammatory mediators and cytokines (cytokine storm), which presented with lymphopenia, high NLR, PLR, and serum CRP, similar to previous studies [29,30,31]. These findings are associated with disease severity and poor outcomes [32,33]. The greater lung and systemic inflammation might need more time to resolve than that in non-severe patients [29]. Skin manifestations such as maculopapular rash and urticaria were reported during active COVID-19 and after hospital discharge [34]. However, the symptoms described in this study were assessed using a patient-reported questionnaire, so we did not explore the details of the skin rash and its relationship with clinical severity during admission.

Two thirds of patients with community-acquired pneumonia had complete CXR resolution at four weeks and the rate of the resolution was inversely correlated with age and number of lobes involved [35], similar to our findings that two thirds of patients with COVID-19 pneumonia had complete CXR resolution at one month. However, CXR in the severe group showed more frequent residual lesions (59.5% vs. 10.3% in the severe and non-severe groups, respectively), among which most of them (19 of 22) were GGO, while others were mixed GGO and consolidation. Only a few patients (3 of 37, 8.1%) were stable or experienced progression, which might be explained by their age and the greater extent of lung inflammation (multi-lobar involvement). These residual lesions might affect respiratory symptoms such as cough and dyspnea, lower lung volume measured by spirometry, lower oxygen saturation, lower physical activity, and poorer HR-QoL.

Sixty patients (57.1%) were evaluated for pulmonary function by spirometry, small airway function by IOS, airway inflammation by FeNO, and exercise capacity by 6-MWT. We found that the value of each spirometric parameter was within the normal limits and not significantly different between the severe and non-severe groups; however, there was a trend of lower lung volume and lower exercise capacity by 6-MWD and exercise desaturation with increased severity of pneumonia, as found in other studies [8,11]. These abnormalities of pulmonary function after COVID-19 pneumonia, including low lung volume, impaired diffusion capacity, and reduced exercise capacity, might be caused by the residual process of inflammation and were correlated with disease severity. These affected quality of life and could improve thereafter [8,10,36].

According to the COVID-19 pathology of the main bronchi and bronchiolar branches, which showed mild, non-specific focal squamous metaplasia and mild transmural lymphocytic and monocytic infiltrates [37], we found evidence of small airway disorder by IOS in thirty percent of our patients (18 of 60), similar to a study in China that showed increased airway resistance (R5 and R20) at one-month follow-up [11]. However, the evidence of small airway disorder between patients and healthy controls was not different, which might be explained by many factors, such as occult airway diseases or the effect of smoking or air pollutants. Eosinophilic airway inflammation also might not be the cause of small airway disorder, as low FeNO levels were described in a study from Finland [38].

The HR-QoL scores, both physical and mental components, were poorer especially in the severe pneumonia group, which may be attributed to the severity of disease and management, which required more aggressive treatment [39] together with the patient’s isolation from their family and society. Although many factors are associated with worsened psychological status during the COVID-19 outbreak, such as lockdown conditions, social restrictions, fear of contamination, and uncertainty about the pandemic [40], only a few patients in our study had anxiety and/or depression, which might be explained by the lower incidence of COVID-19 in Chiang Mai during the time of assessment (late May–June 2021).

The strength of our study is its value as the first study that evaluates the residual symptoms, pulmonary function including small airway function, exercise capacity, and radiograph, together with quality of life and mood disorders, at one month after hospital discharge for COVID-19 pneumonia in Chiang Mai, Thailand. Healthy controls were also enrolled for comparison with severe and non-severe COVID-19 pneumonia. However, this study has some limitations. Firstly, our study included a single-center cohort. The results may differ at other clinical sites. Secondly, COVID-19 pneumonia during the study period was mostly caused by alpha variants (B.1.1.7), which have different clinical manifestations to other variants of more recent concern in Thailand (delta variant, B.1.617.2) [2,41]. Thirdly, some clinical features, pulmonary function, and abnormal chest radiographs require long-term follow-up to clarify the effect of COVID-19 pneumonia in Thailand.

## 5. Conclusions

Adult patients with COVID-19, especially those with clinically severe pneumonia, still have residual symptoms and chest radiographic abnormalities, together with worse quality of life and exercise capacity, at one month after hospital discharge.

## Figures and Tables

**Table 1:** Demographic data of study population (*n* = 130).

Variables	Severe (*n* = 37)	Non-Severe (*n* = 68)	Healthy Control (*n* = 25)	*p*-Value
Demographic data				
Age (year)	44.5 ± 14.5 ^#^	30.8 ± 14.3 *	43.0 ± 9.6	<0.001
Male gender	21 (56.8)	33 (48.5)	12 (48.0)	0.689
Body mass index (kg/m^2^)	31.5 ± 5.9 *^,#^	26.1 ± 6.1	26.1 ± 5.6	<0.001
Co-morbidities				0.099
No	15 (40.5)	42 (61.8)	15 (60.0)	
At least 1 disease	22 (59.5) ^#^	26 (38.2)	10 (40.0)	
Lists of co-morbidities				
Cardiovascular	16 (43.2) ^#^	4 (5.9) *	6 (24.0)	<0.001
Metabolic	2 (5.4)	1 (1.5)	0 (0.0)	0.283
Respiratory	2 (5.4) ^#^	17 (25.0)	4 (16.0)	0.041
Hematology	1 (2.7)	3 (4.4)	0 (0.0)	0.544
Gastrointestinal	1 (2.7)	0 (0.0)	0 (0.0)	0.282
Neuromuscular	0 (0.0)	1 (1.5)	0 (0.0)	0.632
Smoking status				0.049
Current	0 (0.0)	7 (10.3)	4 (16.0)	
Ex-smoker	9 (24.3)	9 (13.2)	1 (4.0)	
Non-smoker	28 (75.7)	52 (76.5)	20 (80.0)	

Note: Data are presented as mean ± SD or *n* (%); *p*-value from ANOVA with Bonferroni adjustment compared across three groups; *, *p* < 0.05 compared to healthy control group from ANOVA with Bonferroni adjustment; ^#^, *p* < 0.05 compared to non-severe group from ANOVA with Bonferroni adjustment.

**Table 2:** Data during hospitalization (*n* = 105).

Variables	Severe (*n* = 37)	Non-Severe (*n* = 68)	*p*-Value
Data during hospitalization			
Duration of symptoms before hospitalization (days) Median (IQR)	5 (3.5, 7.5)	4 (2, 6)	0.073
Vital signs			
Body temperature (°C)	37.6 ± 1.2	36.7 ± 0.8	<0.001
Pulse rate (beats/min)	92.0 ± 16.7	101.0 ± 17.7	0.012
Respiratory rate (breaths/min)	23.3 ± 4.4	20.4 ± 1.3	<0.001
Mean arterial pressure (mmHg)	93.3 ± 12.4	91.8 ±12.5	0.578
SpO_2_ %))	92.1 ± 5.0	96.0 ± 2.5	<0.001
Chest X-ray score, Median (IQR)	13 (5, 21)	2 (2, 4)	<0.001
Chest X-ray pattern			<0.001
Ground glass opacities	11 (29.7)	60 (88.2)	
Consolidation	3 (8.1)	0	
Mixed	23 (62.2)	8 (11.8)	
Chest X-ray distribution			
Lower lobe involvement	36 (97.3)	66 (97.1)	1
Multi-lobar	35 (94.6)	54 (79.4)	0.047
Bilateral	35 (94.6)	52 (76.5)	0.028
Complete blood count on admission			
Hemoglobin (g/dL)	13.8 ± 1.8	13.6 ± 1.9	0.673
Hematocrit (%)	40.8 ± 4.7	40.7 ± 5.3	0.972
White blood count (×10^3^ cells/mm^3^)	6.9 ± 3.1	5.9 ± 2.1	0.105
Neutrophil count (×10^3^ cells/mm^3^)	5.3 ± 3.1	3.6 ± 1.7	0.004
Lymphocyte count (×10^3^ cells/mm^3^)	1.2 ± 0.6	1.9 ± 0.7	<0.001
Platelet count (×10^3^/mm^3^)	221.9 ± 82.8	248.5 ± 84.3	0.123
Neutrophil–lymphocyte ratio, Median (IQR)	4.1 (2.0, 7.6)	1.7 (1.2, 2.6)	<0.001
Platelet–lymphocyte ratio, Median (IQR)	201.2 (114.4, 289.4)	130.0 (97.9, 167.0)	0.002
Biomarker			
CRP (mg/L), Median (IQR)	67.8 (28.8, 122.6)	16.3 (4.3, 61.7)	0.01
D-dimer (ng/mL), Median (IQR)	463 (339, 826)	391 (272, 556)	0.154
Management at admission			
Antiviral therapy			<0.001
Favipiravir	18 (48.6)	62 (91.2)	
Remdesivir	7 (18.9)	2 (2.9)	
Favipiravir + Remdesivir	11 (29.7)	2 (2.9)	
None	1 (2.7)	2 (2.9)	
Antibiotic	30 (81.1)	19 (27.9)	<0.001
Systemic corticosteroid	37 (100)	41 (60.3)	<0.001
Tocilizumab	8 (21.6)	0 (0.0)	<0.001
Hemoperfusion	6 (16.2)	1 (1.5)	0.007

Note: Data are presented as *n* (%), mean ± SD, or median (IQR). Abbreviations: SpO_2_, oxygen saturation via pulse oximeter; CRP, c-reactive protein.

**Table 3:** Symptoms and vital signs at one-month follow-up visit.

Variables	Severe (*n* = 37)	Non-Severe (*n* = 68)	Healthy Control (*n* = 25)	*p*-Value
Symptoms				<0.001
No symptoms	13 (35.1)	42 (61.8)	23 (92.0)	
At least 1 symptom	24 (64.9) *^,#^	26 (38.2) *	2 (8.0)	
List of symptoms				
Fever	3 (8.1) ^#^	0 (0.0)	0 (0.0)	0.021
Cough	12 (32.4) *^,#^	9 (13.2)	0 (0.0)	0.002
Dyspnea	5 (13.5)	5 (7.4)	0 (0.0)	0.145
Wheeze	1 (2.7)	1 (1.5)	0 (0.0)	0.696
Purulent sputum	1 (2.7)	1 (1.5)	0 (0.0)	0.696
Chest pain	4 (10.8)	5 (7.4)	0 (0.0)	0.253
Sore throat	3 (8.1)	3 (4.4)	0 (0.0)	0.326
Rhinorrhea	3 (8.1)	7 (10.3)	1 (4.0)	0.624
Headache	3 (8.1)	4 (5.9)	1 (4.0)	0.797
Muscle pain	5 (13.5)	4 (5.9)	1 (4.0)	0.278
Fatigue	8 (21.6) *^,#^	5 (7.4)	0 (0.0)	0.012
Nausea/vomiting	2 (5.4)	0 (0.0)	0 (0.0)	0.078
Diarrhea	3 (8.1)	2 (2.9)	0 (0.0)	0.227
Anosmia	0 (0.0)	2 (2.9)	0 (0.0)	0.396
Ageusia	2 (5.4)	1 (1.5)	0 (0.0)	0.305
Skin rash	8 (21.6) *^,#^	5 (7.4)	0 (0.0)	0.012
Vital signs				
Temperature (°C)	36.6 ± 0.2	36.6 ± 0.3	36.5 ± 0.3	0.073
Pulse rate (beats/min)	94.3 ± 12.1 *	93.2 ± 11.5 *	83.8 ± 13.7	0.002
Respiratory rate (breaths/min)	19.2 ± 1.1	18.8 ± 1.1	18.3 ± 2.2	0.084
Mean arterial pressure (mmHg)	104.4 ± 9.4 *^,#^	97.1 ± 10.6	96.6 ± 14.2	0.003
SpO_2_ (%)	97.0 ±1.6 *^,#^	97.8 ± 1.3	98.2 ± 0.9	0.001

Note: Data are presented as *n* (%) or mean ± SD; *p*-value from Chi-squared test compared across three groups; *, *p* < 0.05 compared to healthy control group from Fisher’s exact test; ^#^, *p* < 0.05 compared to non-severe group from Fisher’s exact test; *, *p* < 0.05 compared to healthy control group from ANOVA with Bonferroni adjustment; ^#^, *p* < 0.05 compared to non-severe group from ANOVA with Bonferroni adjustment. Abbreviation: SpO_2_, oxygen saturation via pulse oximeter.

**Table 4:** Dyspnea, quality of life, and mood disorders at one-month follow-up visit.

Parameters	Severe (*n* = 37)	Non-Severe (*n* = 68)	Healthy Control (*n* = 25)	*p*-Value
MMRC dyspnea scale, Median (IQR) ^a^	1 (0, 1) *^,#^	0 (0, 0)	0 (0, 0)	<0.001
Classification ^b^				0.007
0	18 (48.6) *^,#^	56 (82.4)	22 (88.0)	
1	16 (43.2)	10 (14.7)	3 (12.0)	
2	2 (5.4)	1 (1.5)	0 (0.0)	
3	1 (2.7)	1 (1.5)	0 (0.0)	
4	0 (0.0)	0 (0.0)	0 (0.0)	
QoL: EQ-5D-5L				
Score (0–1)	0.77 ± 0.17 *^,#^	0.88 ± 0.16	0.89 ± 0.12	0.001
VAS (0–100)	83.5 ± 11.9	83.5 ± 11.3	87.4 ± 9.5	0.289
QoL: SF-36 (0–100)				
Physical functioning	66.2 ± 21.4 *^,#^	82.4 ± 19.9 *	93.6 ± 10.9	<0.001
Role limitations, physical problems	43.9 ± 44.2 *^,#^	77.2 ± 35.2	92.0 ± 20.1	<0.001
Bodily pain	81.8 ± 22.8 ^#^	91.9 ± 12.7	84.8 ± 18.3	0.013
General health perceptions	60.9 ± 23.2 *	64.9 ± 19.8 *	76.8 ± 12.1	0.008
Vitality	63.5 ± 18.9 *	62.1 ± 21.3 *	77.8 ± 9.8	0.002
Social functioning	71.6 ± 26.5 *	79.6 ± 21.8	92.0 ± 13.9	0.002
Role limitations, emotional problems	54.0 ± 43.3 *^,#^	76.4 ± 38.2	96.0 ± 14.6	<0.001
Mental health	73.3 ± 13.9 *^,#^	76.8 ± 15.4 *	84.9 ± 11.1	0.007
Mood disorder: HADS				
Anxiety score, Median (IQR) ^a^	3.0 (1.5, 5.0)	2.5 (1.0, 5.0)	2.5 (1.0, 5.5)	0.887
Score ≥ 11 ^b^	1 (2.7)	2 (2.9)	0 (0.0)	0.692
Depression score, Median (IQR) ^a^	2.0 (1.0, 5.0)	2.0 (1.0, 4.0)	2.0 (0.5, 3.5)	0.68
Score ≥ 11 ^b^	4 (10.8) *	1 (1.5)	0 (0.0)	0.032
Anxiety and/or depression ^b^	4 (10.8)	3 (4.4)	0 (0.0)	0.158
Both anxiety and depression ^b^	1 (2.7)	0 (0.0)	0 (0.0)	0.282

Note: Data are presented as *n* (%), mean ± SD, or median (IQR); ^a^ *p*-value from Kruskal–Wallis test compared across three groups; *, *p* < 0.05 compared to healthy control group from Mann–Whitney U test or ANOVA with Bonferroni adjustment; ^#^, *p* < 0.05 compared to non-severe group from Mann–Whitney U test or ANOVA with Bonferroni adjustment; ^b^, *p*-value from Chi-squared test compared across three groups. Abbreviations.: MMRC, modified medical research council; QoL, quality of life; EQ-5D-5L, Euro Quality of Life—5 dimensions—5 levels; SF-36, 36-item Short-Form Health Survey; HADS, Hospital Anxiety and Depression Scale.

**Table 5:** Laboratory results and chest radiograph at one-month follow-up visit.

Parameters	Severe (*n* = 37)	Non-Severe (*n* = 68)	Healthy Control (*n* = 25)	*p*-Value
Complete blood count				
Hemoglobin (g/dL)	13.4 ± 1.5	13.4 ± 1.7	13.2 ± 1.7	0.845
Hematocrit (%)	40.5 ± 4.0	40.3 ± 4.5	38.5 ± 8.2	0.266
White blood cells (×10^3^ cells/mm^3^)	9.2 ± 6.1 *^,#^	7.2 ± 1.9	6.9 ± 1.9	0.01
Neutrophil (×10^3^ cells/mm^3^)	5.5 ± 3.8 *^,#^	4.0 ± 1.6	3.9 ± 1.4	0.011
Lymphocyte (×10^3^ cells/mm^3^)	2.9 ± 2.1	2.4 ± 0.7	2.3 ± 0.5	0.079
Eosinophil (×10^3^ cells/mm^3^), Median (IQR)	127.3 (57.3, 198.2)	146.5 (81.7, 234.6)	141.4 (105.3, 257.5)	0.355
Platelet (×10^3^/mm^3^)	339.4 ± 110.6 *	307.8 ± 85.2	280.6 ± 54.9	0.036
Neutrophil–lymphocyte ratio	2.0 ± 0.8	1.8 ± 0.7	1.8 ± 0.7	0.274
Platelet–lymphocyte ratio	139.7 ± 55.7	135.5 ± 43.7	128.8 ± 36.9	0.66
Chest X-ray score, Median (IQR) ^a^	2 (0, 2.5) *^,#^	0 (0, 0)	0 (0, 0)	<0.001
Chest X-ray interpretation				<0.001
Improved with complete resolution	12 (32.4)	58 (85.3)		
Improved with residual lesion	22 (59.5)	7 (10.3%)		
Not improved without progression	1 (2.7)	1 (1.5%)		
Not improved with progression	2 (5.4)	2 (2.9%)		

Note: Data are presented as *n* (%), mean ± SD, or median (IQR); ^a^ *p*-value from Kruskal–Wallis H test compared across three groups; *, *p* < 0.05 compared to healthy control group from Mann–Whitney U test or ANOVA with Bonferroni adjustment; ^#^, *p* < 0.05 compared to non-severe group from Mann–Whitney U test or ANOVA with Bonferroni adjustment.

**Table 6:** Spirometry and FeNO at one-month follow-up visit (*n* = 85).

Parameters	Severe (*n* = 31)	Non-Severe (*n* = 29)	Healthy Control (*n* = 25)	*p*-Value
Spirometry				
FVC (L)	3.05 ± 0.82	3.38 ± 0.79	3.44 ± 0.90	0.168
FVC (% predicted) ^a^	88.3 ± 12.8 *	94.6 ± 13.9	102.4 ± 11.9	0.001
z-score of FVC	−0.864 (−1.523, −0.015) *	−0.310 (−1.260, 0.113) *	0.235 (−0.277, 0.789)	0.002
FEV_1_ (L)	2.59 ± 0.68	2.95 ± 0.68	2.91 ± 0.78	0.118
FEV_1_ (% predicted) ^a^	89.7 ± 12.8 *	96.0 ± 14.7	102.9 ± 11.9	0.002
z-score of FEV_1_	−0.450 (−1.451, −0.014) *	−0.294 (−1.235, 0.384)	0.160 (−0.475, 0.923)	0.005
FEV_1_/FVC (%) ^a^	85.2 ± 5.7	87.4 ± 6.1	84.5 ± 3.5	0.107
z-score of FEV_1_/FVC	0.287 (−0.469, 1.197)	−0.101 (−0.465, 0.913)	−0.280 (−0.486, 0.393)	0.481
FEV_1_/FVC < LLN ^b^	1 (3.2)	0 (0)	0 (0.0)	0.414
FEF_25__-__75__%_ (L/s)	3.30 ± 1.13	3.67 ± 1.07	3.44 ± 1.28	0.463
FEF_25__-__75__%_ (% predicted) ^a^	106.3 ± 34.0	105.5 ± 36.4	108.9 ± 25.6	0.923
z-score of FEF_25-75%_	0.250 (−0.828, 1.103)	0.150 (−0.771, 0.596)	0.210 (−0.318, 0.707)	0.695
FeNO				
FeNO (ppb) ^a^	15.6 ± 6.8	15.5 ± 8.8	13.0 ± 7.9	0.411
FeNO > 25 ppb ^b^	3 (9.7)	5 (17.2)	2 (8.0)	0.520

Note: Data are presented as *n* (%) or mean ± SD; ^a^ *p*-value from ANOVA compared across three groups; *, *p* < 0.05 compared to healthy control from ANOVA with Bonferroni adjustment; ^b^, *p*-value from Chi-squared test compared across three groups. Abbreviations: FVC, forced vital capacity; FEV_1_, forced expiratory volume in first second; FEF_25–75%_, forced expiratory flow at 25–75% of FVC; FeNO, fractional exhaled nitric oxide.

**Table 7:** Impulse oscillometry at one-month follow-up visit (*n* = 85).

Parameters	Mean ± SD	Adjusted Mean Difference (95%CI)	*p*-Value
R5 (kPa/L/s)			
Healthy control (*n* = 25)	0.41 ± 0.15	Ref.	
Non-severe (*n* = 29)	0.35 ± 0.12	−0.06 (−0.14, 0.00)	0.068
Severe (*n* = 31)	0.39 ± 0.11	−0.04 (−0.11, 0.03)	0.245
R20 (kPa/L/s)			
Healthy control (*n* = 25)	0.33 ± 0.12	Ref.	
Non-severe (*n* = 29)	0.26 ± 0.10	−0.06 (−0.12, 0.00)	0.045
Severe (*n* = 31)	0.32 ± 0.10	−0.02 (−0.08, 0.04)	0.471
R5-R20 (kPa/L/s)			
Healthy control (*n* = 25)	0.08 ± 0.05	Ref.	
Non-severe (*n* = 29)	0.08 ± 0.04	−0.00 (−0.03, 0.02)	0.591
Severe (*n* = 31)	0.08 ± 0.03	−0.02 (−0.04, 0.00)	0.079
Fres (Hz)			
Healthy control (*n* = 25)	10.2 ± 3.9	Ref.	
Non-severe (*n* = 29)	10.8 ± 2.9	0.73 (−0.97, 2.43)	0.394
Severe (*n* = 31)	12.0 ± 3.1	0.54 (−1.19, 2.27)	0.536
AX (kPa/L)			
Healthy control (*n* = 25)	0.39 ± 0.45	Ref.	
Non-severe (*n* = 29)	0.40 ± 0.45	0.04 (−0.22, 0.29)	0.772
Severe (*n* = 31)	0.57 ± 0.49	0.06 (−0.20, 0.32)	0.651
X5 (kPa/L/s)			
Healthy control (*n* = 25)	−0.07 ± 0.06	Ref.	
Non-severe (*n* = 29)	−0.08 ± 0.06	−0.01 (−0.05, 0.02)	0.436
Severe (*n* = 31)	−0.10 ± 0.07	−0.02 (−0.05, 0.02)	0.31
Small airway disorder(R5-R20 > 0.1 kPa/L/s)			0.801
Healthy control (*n* = 25)	9 (36.0)		
Non-severe (*n* = 29)	8 (27.6)		
Severe (*n* = 31)	10 (32.3)		

Note: Data are presented as mean ± SD or *n* (%); adjusted mean difference using Gaussian regression adjusted for confounding factors including age, BMI, and gender. Abbreviations: R5, resistance at 5 Hz; R20, resistance at 20 Hz; R5-R20, heterogeneity of resistance between R5 and R20; Fres, resonant frequency; X5, reactance at 5 Hz; AX, area under reactance curve between 5 Hz and resonant frequency.

**Table 8:** Exercise capacity and exercise desaturation test at one-month follow-up visit (N = 85).

6-MWD (m)	Mean ± SD	Adjusted Mean Difference (95%CI)	*p*-Value
Healthy control (*n* = 25)	525.5 ± 36.4	Ref.	
Non-severe (*n* = 29)	451.7 ± 78.9	−79.2 (−116.5, −41.8)	<0.001
Severe (*n* = 31)	419.9 ± 74.4	−103.8 (−141.8, −65.7)	<0.001
Exercise desaturation (SpO_2_ decrease ≥ 3%)	*n* (%)	Adjusted risk ratio (95%CI)	*p*-value
Healthy control (*n* = 25)	0 (0.0)		
Non-severe (*n* = 29)	1 (3.2)	Ref.	
Severe (*n* = 31)	6 (19.4)	3.31 (0.38, 28.96)	0.279

Note: Data are presented as *n* (%) or mean ± SD; adjusted mean difference using Gaussian regression adjusted for confounding factors including age, BMI, and gender. Abbreviations: 6-MWD, 6-min walk distance; SpO_2_, oxygen saturation via pulse oximeter.

## Data Availability

The datasets used and/or analyzed during the current study are available from the corresponding author on reasonable request.
